# Impact of Three Waves of the COVID-19 Pandemic on the Rate of Elective Cataract Surgeries at a Tertiary Referral Center: A Polish Perspective

**DOI:** 10.3390/ijerph18168608

**Published:** 2021-08-14

**Authors:** Diana Anna Dmuchowska, Barbara Pieklarz, Joanna Konopinska, Zofia Mariak, Iwona Obuchowska

**Affiliations:** Ophthalmology Department, Medical University of Bialystok, 24a M. Sklodowskiej-Curie, 15-276 Bialystok, Poland; barbara.pieklarz@gmail.com (B.P.); joannakonopinska@o2.pl (J.K.); mariakzo@umb.edu.pl (Z.M.); iwonaobu@wp.pl (I.O.)

**Keywords:** cataract surgery, pandemic, ophthalmic epidemiology, public health, SARS-CoV-2, lockdown, simultaneous bilateral cataract surgery

## Abstract

The aim of this study was to assess the effect of three waves of the COVID-19 pandemic on the number of elective cataract surgeries. A retrospective single-center consecutive case series study was performed. We included all 12,464 patients who received cataract surgery in the period between 1 January 2016 and 31 May 2021. Monthly numbers of cataract surgeries during the pandemic were compared with monthly numbers in the reference years 2016–2019. In the pandemic the number of cataract surgeries decreased by 53.4%. The monthly numbers during the first, second and third wave of the pandemic were 77.5%, 51.5% and 29.7% lower, respectively, compared with the reference level. No rebound effect was observed once the pandemic restrictions were eased. Simultaneous bilateral cataract surgeries (SBCS) constituted 6.5% of cataract procedures performed in April and May 2021 compared with 0.77% carried out between May 2019 and March 2021. While the pandemic-affected monthly numbers of cataract surgeries tend to increase recently, they are still below the prepandemic level. Patients should be encouraged to weigh the risks of COVID-19-related morbidity and mortality against the benefits of cataract surgery. Reorganization of the logistics of cataract services is advisable with consideration of SBCS as one of the options.

## 1. Introduction

Coronavirus disease COVID-19 pandemic has substantially affected not only referrals to ophthalmic emergency departments but also the number of elective ophthalmic procedures conducted worldwide [1,2,3,4,5]. Cataract surgeries had been completely suspended in many countries [6,7]. To tackle the expected COVID-19 cataract backlog [8], a number of strategies for cataract service recovery have been implemented by various centers worldwide. For example, risk stratification and prioritization protocols were adapted in some countries [8,9,10]. Adequate safety measures to decrease the risk of COVID-19 transmission were implemented as well [8,11,12]. Finally, simultaneous bilateral cataract surgery has been advised in some countries, including United States [13], Germany [14], UK and Ireland [8,15]. In Poland, elective cataract surgeries were suspended entirely during the first wave of the pandemic, between 12 March 2020 and 7 May 2020.

Since 1 April 2019, cataract surgeries in Poland are reimbursed by the National Health Fund without any limits, and the only factor defining the number of conducted procedures is the capacity of a given center. As a result, a gradual decline in patient volume was observed on the waiting lists. In that context, Poland differs from other countries. Our center, the Department of Ophthalmology at the Medical University of Bialystok (Białystok, Poland), is a tertiary referral center for adults from Podlaskie Voivodeship (a population of approximately 1.2 million). After the abovementioned change in the reimbursement policy, the waiting time for elective cataract surgery at our center shortened from about four months to approximately 2 weeks in August 2019. Except for clinical criteria, it was mainly the willingness of patients that impacted the quantity of the performed procedures. In general, this situation is representative of other Polish centers as well.

Most previous studies analyzing the impact of the pandemic on the number of cataract surgeries focused on the early effects of the first wave [2,7,14,16]. To the best of our knowledge, the present study is not only the first published comparison of pre- and intrapandemic cataract surgery numbers but also the first analysis of the impact each of the three waves of the pandemic had on the surgical volume. We also verified whether the loosening of pandemic-related restrictions contributed to a rebound effect in the number of cataract procedures. This is the first such report from a Central Eastern European center.

We cannot predict the future course of the pandemic, and the fourth wave is still likely to occur, especially considering the emergence of the new mutations of the coronavirus. Hence, it is important to reconsider current approaches to cataract surgery and prepare for various possible scenarios. The primary aim of this study was to assess the effect of the three waves of the COVID-19 pandemic on the number of elective cataract surgeries. This study provides an insight into the pandemic-related fluctuations in the surgical volume, and its results may facilitate decisions about the organization of pandemic-affected healthcare services.

## 2. Materials and Methods

### 2.1. Study Design, Participants, Eligibility Criteria and Ethics

The study was designed as a retrospective single-center consecutive case series audit for the International Classification of Diseases, Tenth Revision (ICD-10) final diagnosis codes B18 and B19 encompassing cataract removal. The data were extracted for the period between 1 January 2016, and 31 May 2021, when a total of 12,464 patients received cataract surgery at a single tertiary referral center, the Department of Ophthalmology, Medical University of Bialystok (Białystok, Poland). As the primary focus of the study was to assess the numbers of patients (not eyes), simultaneous bilateral cataract surgeries were accounted as single procedures. Simultaneous bilateral cataract surgeries have been performed at our center since May 2019 and based on the decision of local authorities, the frequency of these procedures has increased starting on 1 April 2021.

All elective cataract surgeries performed during the period specified above were included in the audit without any exclusions.

To adjust the analysis for month-to-month fluctuations, the mean monthly number of elective cataract surgeries performed between 1 January 2016 and 31 December 2019 was considered a reference value.

The reference value was compared with the mean monthly number of cataract surgeries during the first 15 months of the COVID-19 pandemic overall, as well with mean monthly numbers of the surgeries during the three waves of the pandemic and two interwave periods.

The first case of COVID-19 in Poland was reported on 4 March 2020, with the first nationwide lockdown imposed on 23 March 2020. A total of three waves of the COVID-19 pandemic have been recorded in Poland thus far, with epidemic restrictions of a various kind being tightened and loosened accordingly. The Polish government introduced and lifted the restrictions according to the number of diagnosed COVID-19 cases, related morbidity and mortality, and capacity of the healthcare system. To analyze the impact of the first 15 months of the COVID-19 pandemic in Poland (from 4 March 2020 to 31 May 2021) on the number of performed elective cataract surgeries, the mean monthly number of the surgeries during this period was compared with the reference level. Additionally, mean monthly numbers of cataract surgeries during the three waves of the COVID-19 pandemic in Poland were analyzed using the dates of introducing and loosening of epidemic restrictions as the cut-off points [17]. The first wave was defined as a period between diagnosing the first COVID-19 case on 4 March 2020 and lifting most restrictions on 31 May 2020. The second wave corresponded to a period between a sudden increase in the number of new COVID-19 cases on 1 October 2020 and loosening most related restrictions on 31 December 2020. The third was defined as a time from another sudden increase in the number of new COVID-19 cases on 1 March 2021 to a gradual loosening of related restrictions on 30 April 2021. Mean monthly numbers of elective cataract surgeries during these three periods were compared with the reference level.

The first interwave period spanned from 1 June 2020 to 30 September 2020, and the second from 1 January 2021 to 28 February 2021. Based on the analysis of mean monthly numbers of elective cataract surgeries during those periods, we verified whether the lifting of epidemic restrictions was associated with a rebound effect on the surgical volume.

The protocol of the study followed the provisions of the Declaration of Helsinki. It was approved by the Local Bioethics Committee at the Medical University of Bialystok as a quality assurance project (decision no. APK.002.87.2021). A waiver of informed consent was approved by the Local Bioethics Committee, given the retrospective nature of the study.

### 2.2. Procedure/Proceedings

Aside from the visit on the operation day, one preoperative appointment and two follow-up visits (1 and 14 days after the surgery) are scheduled for each patient treated at our center. Phacoemulsification with intraocular lens implantation is performed as the same-day procedure. In selected cases, simultaneous bilateral cataract surgery is performed. An additional visit was added to this protocol during the COVID-19 pandemic. Starting on 7 May 2020, each patient must undergo preoperative COVID-19 screening with a polymerase chain reaction (PCR) testing 2 days before the surgery and then stay under self-isolation until the procedure. The cost of preoperative test is fully covered by the National Health Fund. Since 1 April 2021, patients no longer need to be tested within 3 months after a positive result of the COVID-19 test or after being vaccinated.

Since 7 May 2020, 25 (0.02%) asymptomatic patients tested positive for COVID-19 and their cataract surgeries were postponed for 6 weeks. A total of three patients tested positive for COVID-19 in April and May 2021. Such a low number of positive patients is unlikely to bias the results of this study significantly.

### 2.3. Statistical Analysis

Statistical analyses were carried out with R software, version 4.0.5 (http://cran.r-project.org (accessed on 13 August 2021)). Sensitivity analysis for change in number of elective cataract surgeries was performed with calculation of % change in number of surgeries vs. corresponding period in 2019.

## 3. Results

### 3.1. The Impact of the COVID-19 Pandemic on the Number of Elective Cataract Surgeries during the First 15 Months after the Outbreak

**Figure 1:**
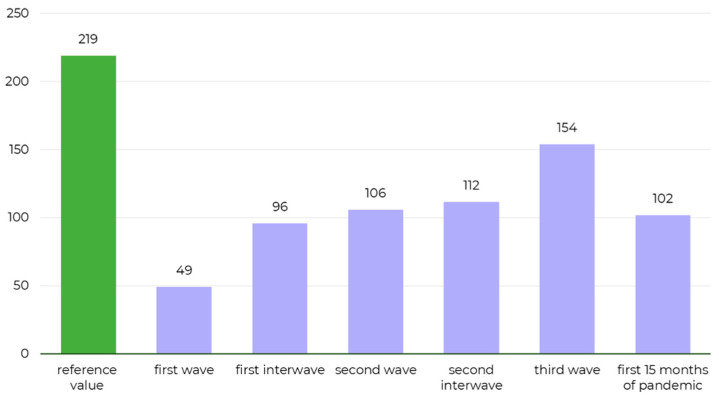
Bar chart showing mean monthly numbers of elective cataract surgeries performed during the analyzed periods. Timespans as described in the Material and Methods section. The reference level was calculated as a mean monthly number of cataract surgeries in 2016–2019.

**Figure 2:**
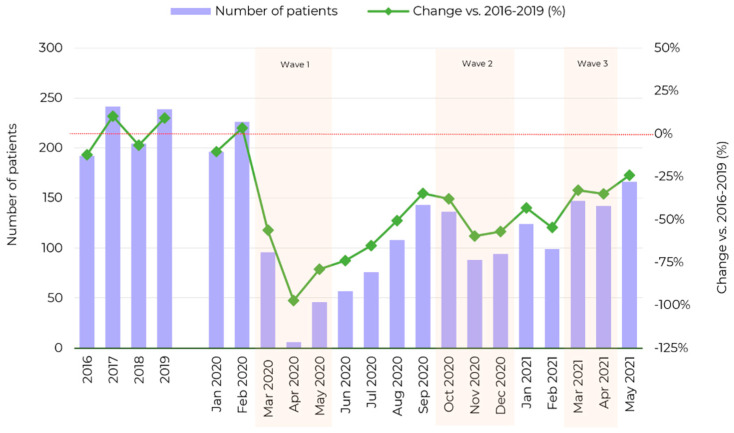
Bar chart showing mean monthly numbers of patients undergoing elective cataract surgery performed during the analyzed periods. The reference level was calculated as a mean monthly number of cataract surgeries in 2016–2019 (raw data in Table S1).

Monthly numbers of patients undergoing cataract surgeries since the outbreak of the COVID-19 pandemic (mean *n* = 101.9) are presented in Figure 1 and Figure 2, along with the reference values (mean monthly numbers of cataract surgeries in 2016–2019, *n* = 219). Overall, the COVID-19 pandemic contributed to a 53.4% decrease in the number of performed elective cataract surgeries.

Simultaneous bilateral cataract surgeries constituted 6.5% (20/308) of all cataract procedures performed in April and May 2021 compared with merely 0.77% (27/3503) of all surgeries carried out between May 2019 and March 2021.

### 3.2. The Impact of Three Waves of the COVID-19 Pandemic on the Number of Elective Cataract Surgeries

Mean monthly numbers of elective cataract surgeries during the first, second and third wave of the pandemic were 77.5%, 51.5% and 29.7% lower, respectively, compared with the reference level of 219 surgeries monthly in 2016–2019 (Figure 1 and Figure 2). While a gradual, steady increase was observed after the first wave of the pandemic, the number of surgeries did not reach the reference level. The numbers of cataract surgeries in March, April and May 2021 were relatively stable. The mean monthly number of the surgeries during the first interwave period (*n* = 96) was 13.9% lower than the mean monthly number during the second interwave period (*n* = 112). No rebound effect was observed in the number of cataract surgeries. The number of cataract surgeries has been steadily increasing since the first wave (*n* = 49). The second (*n* = 106) and third waves (*n* = 154) did not cause a decrease in the number of surgeries in comparison to the interwave periods (*n* = 96 and *n* = 112, respectively). Sensitivity analysis confirmed the drop in number of surgeries in each wave vs. corresponding period from 2019—a decline of 85.0%, 38.0% and 53.8% for first, second and third wave of the pandemic, respectively (detailed description in Table S2, Figure S1).

## 4. Discussion

This study demonstrated that the COVID-19 pandemic contributed to an evident, albeit variable in time, decrease in the number of performed elective cataract surgeries. Despite a recent gradual increase, the number of procedures still did not reach its prepandemic level (as of 31 May 2021). The mean monthly number of cataract surgeries performed at our center decreased by 53.4% compared with 2016–2019. This observation stays in opposition to the results of a study from the UK in which 83.3% of patients were willing to undergo cataract surgery despite the risks of contracting COVID-19 while travelling and attending hospital [7]. However, the difference in the waiting time (about 18 weeks in the UK [18] vs. a few days in our department) needs to be considered. Furthermore, due to the study design (audit and not casesheets analysis) we were not able to verify whether demographic and clinical characteristics of patients with cataract influenced their willingness to undergo cataract surgery during the COVID-19 pandemic.

The etiology of the decrease in the number of cataract surgeries performed at our center may be complex, involving problems with transportation, limited access to outpatient clinics, and patients’ desire to minimize a nonessential exposure to COVID-19 [19]. Another factor that might potentially limit patient’s referrals was obligatory preoperative COVID-19 test performed 2 days before the surgery and followed by self-isolation. Despite the fact that cost of the preoperative test was covered by the National Health Fund, it still required a prolonged visit (if same-day with preoperative assessment) or additional visit (if on separate days). However, the legislation has recently changed, and starting on 1 April 2021, fully vaccinated persons and patients with a history of COVID-19 are exempted from the preoperative test. Aside from the legally bound restrictions, patients’ reluctance to visit a hospital and undergo surgery might also be driven by a fear of COVID-19. Patients with cataract treated at our center are primarily older persons with multiple comorbidities, and hence, have an increased risk of COVID-19-related mortality [20]. According to Docherty et al., mortality risk due to COVID-19 increased 8.5-fold in patients between 70 and 79 years of age and 11.1-fold in persons aged 80 years or older [21].

The recently observed gradual increase in the number of referrals for cataract surgeries might be related to the growing vaccination rate and the resultant reduction of the COVID-19 fear [22]. The proportion of partially or fully vaccinated population in Poland increased from 5.8% at the beginning of the third wave of the pandemic (1 March 2021) to 22.6% at the end of April 2021 and 36.1% on 31 May 2021 [23]. Since 9 May 2021, all adults in Poland are allowed to register for the vaccination.

A decrease in the number of performed cataract surgeries is with no doubt an alarming finding. Visual impairment due to cataracts can result in increased mortality and risk of depression [24,25,26]. Cataracts may also have a social impact, for example, by affecting the ability of self-care and driving skills [5]. Improved vision after cataract surgery was in turn shown to be associated with better quality of life and lesser risk of falls, hip fractures and dementia [26,27,28].

Tognetto et al. proposed a safe and efficient strategy to reorganize cataract surgery in the pandemic era [5]. The changes proposed by those authors involved a preoperative, perioperative and postoperative period. The postulated changes included reducing the visit number and time spent by the patient in a hospital setting through the introduction of the same-day preoperative assessment and testing or same-day preoperative assessment and surgery. Additionally, the reduction of postoperative follow-up was advised [5]. Unfortunately, the implementation of the latter change in Poland would require modifying regulations at the national level. Another optimization strategy, already implemented at our center, is an increase in the proportion of simultaneous bilateral cataract surgeries. Several authors suggested that in the pandemic era, such an approach seems to be more applicable than ever [8,13,14,15]. The benefits include reduced COVID-19 exposure, lower costs, lesser number of visits and decrease in work absenteeism [13,29,30].

In the Polish context, it is essential to estimate whether the surgical volume would return to normal or be still reduced. The first scenario would require the implementation of increased safety measures, which translates into the longer time allocated to a single patient and involvement of additional medical, nursing and administrative personnel. Until now, the use of these extra resources has been compensated by reduced demand for cataract surgeries. We presume that due to postponing their surgery, the patients will present with more advanced cataract with all the consequences from both patient and surgeon perspective. Additionally, the reduced surgical volume impacts the residents’ training program [31,32]. Fewer opportunities for acquisition and improvement of their surgical skills rises a concern about their qualifications and patient care in the future, especially if such situation continues. From the economic point of view, in the longer term, our department should make up for the backlog in the number of cataract surgeries. The terms of the contract with the National Health Fund are briefly described in Figure S1. Encouraging informative actions about safety procedures against COVID and advantages/disadvantages of the surgery itself may need to be implemented.

One potential limitation of this study stems from the fact that it analyzed the changing patterns of presentations for elective cataract surgeries solely in a Polish context. Hence, the results cannot be generalized to other countries. However, analysis of a specific epidemiological situation in a particular country may provide information on the effects of the country’s pandemic strategy. Due to the study design (audit and not casesheets analysis) we were not able to verify whether demographic and clinical characteristics of patients with cataract influenced their willingness to undergo cataract surgery during the COVID-19 pandemic.

This study was not only the first to compare pre- and intrapandemic cataract surgery numbers but also the first published analysis of the impact each of the three waves of the pandemic had on the surgical volume. Due to such an approach, we could obtain a better insight into the relationship between the dynamics of the pandemic and fluctuations in the number of elective cataract surgeries. The results were confirmed by the sensitivity analysis.

## 5. Conclusions

In Poland, a decrease in the number of elective cataract surgeries was observed not only during the three waves of the COVID-19 pandemic but also during the interwave periods when the epidemic restrictions were eased. While monthly numbers of the surgeries tend to increase recently, they are still below the prepandemic level. Patients should be encouraged to weigh the risks of COVID-19-related morbidity and mortality against the benefits of cataract surgery. Educational activities and information campaigns need to be implemented to enlighten the patients about the safety measures introduced during cataract surgeries in the pandemic era. Additionally, cost of the logistics of cataract services is advisable with consideration of simultaneous bilateral cataract surgeries. This study provides an insight into the pandemic-related fluctuations in cataract surgery volumes and adds to a global discussion about the impact of COVID-19 on ophthalmic procedures; hence, its results may facilitate decisions about the organization of pandemic-affected healthcare services.

## Data Availability

Readers can access the data supporting the conclusions of the study upon email request. The names and personal data of the participants cannot be released, due to ethical aspects.

## Supplementary Materials

The following are available online at www.mdpi.com/article/10.3390/ijerph18168608/s1, Figure S1: Bar chart showing mean monthly numbers of patients undergoing elective cataract surgery performed between January 2019 and May 2021, Table S1: Change in numbers of elective cataract surgeries performed during the analyzed periods vs. reference value, Table S2: Sensitivity analysis for change in numbers of elective cataract surgeries performed during the analyzed periods vs. corresponding periods in 2019.
