# Supplementary material for: Impact of Three Waves of the COVID-19 Pandemic on the Rate of Elective Cataract Surgeries at a Tertiary Referral Center: A Polish Perspective
Source: Int J Environ Res Public Health. 2021 Aug 14;18(16):8608. doi: 10.3390/ijerph18168608 (PMC8393808; doi:10.3390/ijerph18168608)
Supplement: Supplementary file 1 [file ijerph-18-08608-s001.zip › final Figure S1 (2).pdf]

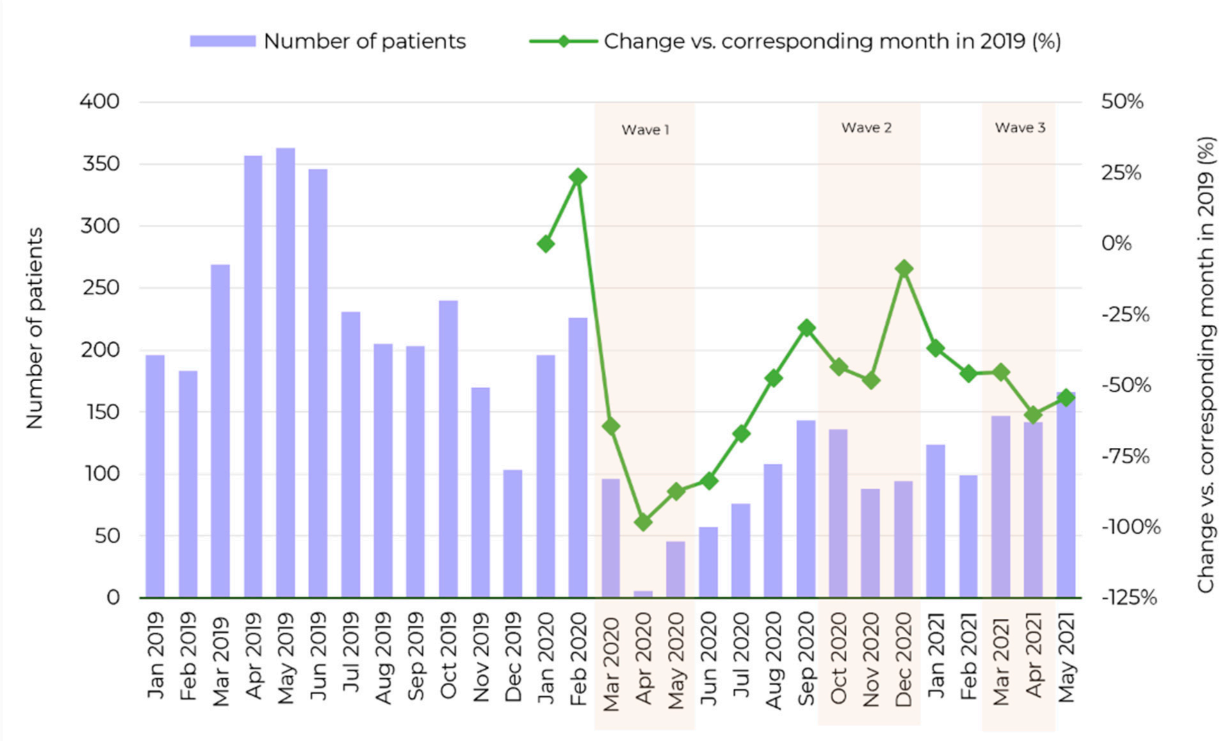

Figure S1. Bar chart showing mean monthly numbers of patients undergoing elective cataract surgery performed between January 2019 and May 2021. Green line represents % change in number for a given month vs. corresponding month in 2019 with reference on the right axis (e.g. -48.2% for Nov 2020 means that number of operated patients was 48.2% lower than in Nov 2019) (raw data and sensitivity analysis in Table S2).

Notes: According to the contract with the National Health Trust the mean of 200 cataract surgeries per month should be performed in the Ophthalmology Department of the Medical University of Bialystok. On top of that additional surgeries performed in afternoons and on weekends were allowed and covered in (usually in April, May and June as in 2019). In 2016-2019 these terms were respected. However, during the pandemic the surgical volume did not reach even 200 procedures per month so the additional surgeries were not performed at all.
