# Supplementary material for: Impact of Three Waves of the COVID-19 Pandemic on the Rate of Elective Cataract Surgeries at a Tertiary Referral Center: A Polish Perspective
Source: Int J Environ Res Public Health. 2021 Aug 14;18(16):8608. doi: 10.3390/ijerph18168608 (PMC8393808; doi:10.3390/ijerph18168608)
Supplement: Supplementary file 1 [file ijerph-18-08608-s001.zip › final Table S1 (2).pdf]

Table S1. Change in numbers of elective cataract surgeries performed during the analyzed periods vs reference value. The reference level was calculated as a mean monthly number of cataract surgeries in 2016-2019 (n=219).

Period	Number of patients/month*	Change vs. reference level
2016	191,8	-12%
2017	241,4	10%
2018	204,1	-7%
2019	238,8	9%
Reference value	219	0%
Jan 2020	196	-11%
Feb 2020	226	3%
Mar 2020	96	-56%
Apr 2020	6	-97%
May 2020	46	-79%
Jun 2020	57	-74%
Jul 2020	76	-65%
Aug 2020	108	-51%
Sep 2020	143	-35%
Oct 2020	136	-38%
Nov 2020	88	-60%
Dec 2020	94	-57%
Jan 2021	124	-43%
Feb 2021	99	-55%
Mar 2021	147	-33%
Apr 2021	142	-35%
May 2021	166	-24%

*mean for each year of period 2016-2019
