# Supplementary material for: Impact of Three Waves of the COVID-19 Pandemic on the Rate of Elective Cataract Surgeries at a Tertiary Referral Center: A Polish Perspective
Source: Int J Environ Res Public Health. 2021 Aug 14;18(16):8608. doi: 10.3390/ijerph18168608 (PMC8393808; doi:10.3390/ijerph18168608)
Supplement: Supplementary file 1 [file ijerph-18-08608-s001.zip › final Table S2 (2).pdf]

Table S2. Sensitivity analysis for change in numbers of elective cataract surgeries performed during the analyzed periods vs corresponding periods in 2019.

Period	Number of patients	Change vs. corresponding period in 2019 (%)
Jan 2019	196	
Feb 2019	183	
Mar 2019	269	
Apr 2019	357	
May 2019	363	
Jun 2019	346	
Jul 2019	231	
Aug 2019	205	
Sep 2019	203	
Oct 2019	240	
Nov 2019	170	
Dec 2019	103	
Jan 2020	196	0,0%
Feb 2020	226	23,5%
Mar 2020	96	-64,3%
Apr 2020	6	-98,3%
May 2020	46	-87,3%
Jun 2020	57	-83,5%
Jul 2020	76	-67,1%
Aug 2020	108	-47,3%
Sep 2020	143	-29,6%
Oct 2020	136	-43,3%
Nov 2020	88	-48,2%
Dec 2020	94	-8,7%
Jan 2021	124	-36,7%
Feb 2021	99	-45,9%
Mar 2021	147	-45,4%
Apr 2021	142	-60,2%
May 2021	166	-54,3%
COVID wave 1 (Mar-May 2020)	148	-85,0%
COVID wave 2 (Oct-Dec 2020)	318	-38,0%
COVID wave 3 (Mar-Apr 2021)	289	-53,8%

Calculation of % change in each month / wave was conducted vs. corresponding period in 2019 (this approach excluded any impact of seasonality)
